# Visual hallucinations and epilepsy: a case report.

**DOI:** 10.1192/j.eurpsy.2026.11333

**Published:** 2026-07-31

**Authors:** G. Lorenzo Chapatte, M. Ríos Vaquero, A. Monllor Lazarraga, L. Rojas Vázquez, M. P. Pando Fernández, L. Rodríguez Andrés, G. Guerra Valera, L. Sobrino Conde, M. D. L. Á. Guillén Soto, A. Aparicio Parras

**Affiliations:** 1 Psiquiatría; 2 Psicología, Hospital Clínico Universitario Valladolid, Valladolid, Spain

## Abstract

**Introduction:**

Visual hallucinations are a clinically significant presentation in emergency settings, warranting careful consideration within both psychiatric and neurological domains. While they may arise in the context of primary psychotic disorders, toxic-metabolic states, or acute confusional syndromes, they are also a recognized manifestation of temporal lobe epilepsy. In this context, visual hallucinations can occur as part of the aura or during focal impaired-awareness seizures, offering valuable insights for the localization and characterization of epileptogenic networks. Accurate recognition and interpretation of these phenomena in the acute setting are essential to inform the differential diagnosis, ensure timely intervention, and guide the development of an appropriate therapeutic strategy.

**Objectives:**

To address the importance and multidisciplinary approach of the presence of visual hallucinations in patients in the psychiatric emergency based on the presentation of the aforementioned clinical case.

**Methods:**

Bibliographic search and description of a clinical case of a patient under follow-up by emergency psychiatry at the “Hospital Clínico Universitario de Valladolid”.

**Results:**

A 55-year-old male from Madrid was admitted to the Emergency Department with generalized seizures without loss of consciousness, which resolved after benzodiazepine administration. In the preceding days, he had experienced multiple episodes of structured visual hallucinations, causing significant distress. After excluding substance intoxication and acute organic pathology, his history of temporal lobe epilepsy, diagnosed in adolescence and under neurological follow-up, was considered relevant. Psychiatric history included mixed adaptive disorder and binge eating disorder, without psychosis, schizophrenia, or bipolar disorder. The visual hallucinations, in combination with the seizure episode, were likely related to his temporal lobe epilepsy diagnosis.

**Conclusions:**

Visual hallucinations in the emergency setting are a clinically significant finding with potential psychiatric or neurological origins, particularly temporal lobe epilepsy. Timely recognition requires a comprehensive evaluation supported by a detailed clinical history, which is essential to guide the differential diagnosis and prevent misclassification. This approach is fundamental to optimize acute management, define appropriate therapeutic strategies, and improve patient outcomes.

**Disclosure of Interest:**

None Declared

